# Becoming the Metalinguistic Mind: The Development of Metalinguistic Abilities in Children from 5 to 7

**DOI:** 10.3390/children9040550

**Published:** 2022-04-13

**Authors:** Sergio Melogno, Maria Antonietta Pinto, Marco Lauriola

**Affiliations:** 1 Department of Developmental and Social Psychology, “Sapienza” University of Rome, Via dei Marsi, 78, 00185 Rome, Italy; mariantonietta.pinto@uniroma1.it (M.A.P.); marco.lauriola@uniroma1.it (M.L.); 2 Faculty of Psychology, “Niccolò Cusano” Telematic University of Rome, Via Don Carlo Gnocchi, 3, 00166 Rome, Italy

**Keywords:** metalinguistic development, metalinguistic tasks, cognitive development, typical development, 5–7-year-old children

## Abstract

The object of this study is the development of metalinguistic abilities in an age range—5 to 7 years—where an important turn takes place in education, namely the transition between kindergarten and primary school. Based on the literature starting from the 70’s of the last century, embryonic forms of awareness of how language variation can be manipulated to convey variation in meaning are widely attested in preschoolers. These forms, however, denote an intuitive and implicit level of awareness and will attain a “meta-level”, based on more systematic and explicit reflectiveness, later in development in correlation with cognitive, linguistic, and educational factors. To measure the development of these abilities across the above age range, we recruited 160 native Italian-speaking children from 5 to 7, with comparable numerosity at each age, gender balance, average socio-cultural background, and no cognitive nor neuropsychological impairment. We used 6 metalinguistic tasks, the Raven’s CPM, a lexical and grammatical ability tests. The results showed a significant increase in all the measures across the span considered and correlations between all the measures. A factor analysis on the metalinguistic tasks showed that a single factor accounted for a large part of the common variance.

## 1. Introduction

The study of metalinguistic abilities, defined as “the ability to reflect upon and manipulate the structural features of spoken language”, to mention a widespread citation [1], has stimulated the attention of scholars from different disciplines, spanning from general to applied linguistics, cognitive psychology, general and developmental psycholinguistics, across the last five decades (for a review, see [2,3]). In more recent times, Ramirez and colleagues [4], modulated the above definition of metalinguistic awareness as “the ability to distance oneself from the content of speech to reflect upon and manipulate the structure of language”.

The adjective “metalinguistic” and the related substantive “metalanguage” were first coined by a neo-positivist philosopher, Carnap [5] to designate a language whose aim was to describe language itself, hence the use of the Greek prefix “meta” which means “beyond”, “upon”, in this case, “beyond” and “upon” language. As pointed out in Pinto & El Euch’s review [2], the term “metalinguistic” has later been adopted by linguist Jakobson [6], to define one of the six functions of language, namely the metalinguistic function, which requires attention to the structural features of the linguistic code per se. Interestingly, Jakobson suggested that this kind of attention is constantly at work in our everyday linguistic exchanges, in comprehension as well as in production, and provides a strong basis for any form of language acquisition, from mother tongue to additional languages. During the seventies of the last century, the attentional processes underlying metalinguistic awareness have become an important object of study for psychologists, who saw the gradual emergence of metalinguistic abilities as a relevant point for both language acquisition and metacognitive growth. In parallel to the early studies on the development of metacognition in children [7,8,9], several experimental works were implemented exploring various types of metalinguistic abilities, from preschool to school age, as well as studies based on observation in natural contexts (For a review on this period, see Hakes; Tunmer, Pratt, and Herriman [1,10]. On the one hand, it soon appeared that children as young as 2.6 years old could show very precocious forms of grammatical or phonological awareness, such as the capability to correct and re-direct their utterances [11], or, just one year later, to identify rhymes on a spontaneous basis or in experimental tasks [12,13,14]. On the other hand, the development of single metalinguistic abilities [15], as measured through formal tasks, such as detecting words in an utterance, defining words, detecting and editing incorrect grammatical forms, detecting phonological units, and grasping their relation to differences in meaning, or detecting pragmatic ambiguities in a message, appeared more gradual.

Gombert’s [16] systematic review of these studies focused on separate areas, namely metaphonological, metasyntactic, metalexical and metasemantic, metapragmatic, and metatextual, showed that children were not able to adequately face the tasks assessing the corresponding abilities before the age of 6 or 7, to the exception of some successful cases. already at the age of 5. For instance, in a classical acceptability task, like in James and Miller’s study [17], children were requested to indicate in a list of sentences those that were incorrectly built, such as “the large rock walked down the hill” (subject-verb rule violation) or “the happy pencil walked down the hill” (adjective-noun rule violation). Children were able to indicate the incorrectly built sentences as young as 5-years-old. In the metasemantic area, in Berthoud-Papandropoulou’s study [18], children were requested to define what is a word, and give examples of words with particular characteristics. a task that involves the capability to separate form from referents. Children under 5 were generally incapable of defining the term ‘word’ and the examples they provided prevailingly concerned objects or animals. For instance, when asked how one can know something is a word, a child replied: “Because you can see what it is”. When asked to give an example of a long word, children most often indicated long objects like ‘train’ ‘because it has many carriages’, and when asked to give an example of a difficult word, they most often indicated a difficult action (ex: “someone who takes away the key…because it’s difficult”). In all the above cases, children of that age seemed unaware of the basic distinction between the physical world and the world of language. By contrast, 5- to 6-year-olds and many of the 6- to 7-year-olds defined the word as being the act of speaking itself (“a word is something you say”). At age 6–7, some children started to consider words as labels, composed of single letters, distinct from physical objects, although they rejected articles, prepositions, or pronouns as words, because “they do not have many letters”. The following paradox is illuminating in this respect: a child refused to acknowledge the pronoun ‘she’ as a word because of insufficient letters but acknowledged the French word âne (‘donkey’) as a word, also composed of only three letters, because “it exists”. Finally, it is not before the age of 7 that children started viewing words as pertaining to a specific linguistic plane (ex: “It’s a piece of a story”; “You have a sentence, and there are words in it”), and sometimes defined them using a grammatical terminology (Ex: “a word is a noun, an adjective, a verb…”).

To account for developmental differences in performance, certainly related to the degree of complexity and formality of tasks, Gombert [16], resorted to a conceptual distinction created by linguist Culioli [19], between *epilinguistic* and *metalinguistic* awareness. The former refers to cognitive processes that address the structural features of language on an intuitive basis, generally not verbalized or little articulated, while the latter refers to attentional processes deliberately focused on these features, consciously verbalized and explained. In the adult, this distinction is not to be intended as clear-cut because there may be constant transitions between degrees or levels of awareness in a very short lapse of time [20]. In the developing child, instead, the transition from epilinguistic to metalinguistic processes takes place during a much longer period, and represents a step forward toward a more abstract way to process language, a real turn toward ‘a metalinguistic mind’. This turn involves the capability to overcome some cognitive conflicts, typically those that sometimes occur between form and content. For instance, if we ask a preschooler if the word “blueberry” is long or short, he/she will have to distinguish between the characteristics of the content—in this case, the limited size of the blueberry—and the characteristics of the form of the word, which is a long one. The capability to make such a distinction requires abstraction from content, and thus represents a significant passage towards a meta-level.

In the literature on metalinguistic awareness, the epi/metalinguistic distinction is more commonly treated as a distinction between implicit and explicit forms. The model of metalinguistic development created by Karmiloff-Smith [21], called Representational Redescription (RR), specifies four steps children must take to pass from implicit to explicit language awareness. In general terms, RR is defined as “…a process by which implicit information in the mind subsequently becomes explicit knowledge to the mind, first within a domain, and sometimes even across domains.” [21] (pp. 17–18). Concerning the representation of language, Level 1 is dependent upon external information, in this case, provided by adult models, and culminates in the mastery of separate language microsystems at a purely behavioral, and therefore implicit level. A second level is reached when the child starts to redescribe some mappings between form and content, although this redescription may bring to a transitory phase of incorrect performance. This is termed “Level 1 of explicit representation” (E1), where redescription can only be inferred indirectly and is not fully conscious. Access to consciousness is possible at levels E2 and E3, as a response to pressures from both external information and internal drive towards coherence. The child becomes able to redescribe in explicit, verbal form (more or less exhaustively, which accounts for the difference between levels E2 and E3), the relationships between linguistic units within a given linguistic system (for instance, the relationships between the usages of indefinite and definite articles in the French language).

In another well-known model of metalinguistic development, elaborated by Bialystok [22,23], the levels of awareness are related to the existence of two major components of language processing, termed ‘analysis of knowledge’ and ‘control’, which the author considers as omnipresent in any language use, from the first child’s oral utterances to the most sophisticated written forms of the adult. Analysis is responsible for the representation of linguistic units, in both form and content, while control relies on various forms of attentional strategies to distinguish between form and content. The two components intersect each other as two Cartesian axes, and each component can be implemented at different degrees of complexity and awareness during the whole lifespan, as a function of the cognitive demands posed by education and professional life. In this respect, the school offers instruments to develop literacy skills but, at the same time, demands to perform those skills at increasingly complex levels of analysis and control or with a particular balance of each component. Later in adult life, different jobs will require different balances between levels of analysis and control. For instance, a disk jockey will need a high level of control but a low level of analysis while a lecturer or a simultaneous translator will need high levels of both components [22] (p. 17). To reach the metalinguistic level, Bialystok [22,23], points to three criteria: (a) a relatively high demand on the analysis component; (b) a relatively high demand on the control component; (c) that the object of reflection is some structural feature of language. Overall, in Bialystok’s theoretical framework [24] the general notion of ‘metalinguistic awareness’ is transformed into an operational construct, especially for what concerns control [24]. The nature of this component has been extensively explored in various forms of executive function, such as attentional strategies, inhibition, shifting, and working memory. For instance, in the above-mentioned Word length task [18], focusing on form, separately from the referent, requires inhibiting the representation of a prepotent cognitive response, switching from referent to form, maintaining form in working memory to analyze it according to the length criterion. All these processes require a notable amount of attentional resources.

In developmental terms, it is to be noted that neither Karmiloff-Smith’s nor Bialystok’s models point to precise ages or stages but only to phases or general trends. In this respect, the ages of 5 and 6 are viewed by many authors as a critical turn, as pointed out by Duncan and colleagues [25], themselves mentioned by Altman and colleagues [26]: “Metalinguistic awareness requires the speaker to focus on the structure and form of the language and develops in later stages of language acquisition around the age of 5–6, building on earlier linguistic knowledge” (p. 3). However, while for Karmiloff-Smith advances in language awareness mainly rely on endogenous factors, Bialystok stresses the relevance of the cognitive demands intrinsically related to social experiences, starting from the early steps of the schooling process [21,22,23].

Aside from these conceptual aspects, another strand of research on metalinguistic development has privileged methodological aspects, often proposing other presentation modalities [27,28,29,30]. For instance, concerning lexical segmentation tasks, some authors have distinguished between offline e online tasks [29]. The former refers to implicit knowledge (know-how) while the latter refers to explicit knowledge (know that), i.e., the representation of the process. However, Veldhuis e Kurvers [31] propose a less clear-cut distinction between offline and online tasks. For instance, these authors consider the ‘tapping task’ as offline. The child is only requested to repeat a sentence and indicate how many words it includes using small blocks, without any metalinguistic judgment. Instead, the ‘click task’ may be considered online. The child listens to a recorded sentence where some ‘clicks’ have been inserted (ex.: “The plate in the”–*click*–“cupboard is dirty”) then he/she must repeat it and also re-evoke the click in the exact position. An intermediate case is the ‘dictation task’ where the child narrates some experience (a trip, for instance) and then dictates it to the examiner piece by piece.

Although most studies on metalinguistic abilities addressed typical developmental trajectories, there is also a wide literature on atypical trajectories consisting of neurodevelopmental disorders, such as language disorders [32,33,34,35] and autism spectrum disorders [36,37].

In this study, our aims were:

(1) To measure the growth of metalinguistic abilities in the age range 5–7 where an important turn takes place in education, namely the transition between kindergarten and primary school, as well as in metacognitive growth, as underlined by various authors [1,10,23]. We expected to find a significant increase in successful responses to the metalinguistic tasks chosen to measure this development.

Given the relevance of the acquisition of literacy skills in relation to the evolution of metalinguistic abilities [38,39], we expected the transition from 5 to 6 years, which matches the passage from kindergarten to the first year of primary school, would yield more significant improvement than the passage from 6 to 7, at least for some of the metalinguistic abilities we measured.

(2) To explore the extent to which the metalinguistic abilities chosen for this study were related to one another, beyond their diversity. The hypothesis was to find positive correlations between all the metalinguistic task scores, suggesting the idea of common cognitive processes in the way children cope with the metalinguistic demands involved in these tasks.
(3) To explore the associations between metalinguistic abilities, basic language abilities, and nonverbal intelligence. The hypothesis was to find positive correlations between the scores of the instruments measuring each of these constructs.

## 2. Materials and Methods

### 2.1. Participants

One hundred and sixty children from 5- to 7-year-old participated in this study.

Children’s age ranged from 5 years and 0 months to 7 years and 11 months (M = 76.75 months; SD = 10.01 months) and were clustered into three age groups (Table 1): (1) 5 years and 0 months to 5 years, 11 months, and 30 days (referred to as 5–6 years); (2) 6 years and 0 months to 6 years, 11 months, and 30 days (referred to as 6–7 years); and (3) 7 years and 0 months to 7 years, 11 months, and 30 days (referred to as 7–8 years). The sample included 73 girls, 85 boys, and two participants with missing gender information. Girls and boys were almost equally distributed across age groups (Table 1), and the two variables were uncorrelated (Chi^2^ = 2.21; df = 3; *p* = 0.331).

In terms of school attendance, all the 5–6 years-old were enrolled in the last year of kindergarten, while all the 6–7 years-old were enrolled in grade 1 and all the 7–8 years-old in grade 2 of primary school. In all cases, the institutes were public schools in Rome (Italy), with comparable pedagogical programs. The socio-economic background of the participants’ families, based on both parents’ educational level and profession, could be defined as average. All the children were native speakers of Italian, without learning disabilities based on teachers’ judgment, and adequate overall school performance according to the school year attended. This study did not consider children previously diagnosed with some impairment or neurodevelopmental disorder. The teachers were required to ask the participants’ families to consent to this study in oral form, which was obtained for all the children. The research design was approved by the Ethical Committee of the Department of Developmental and Social Psychology-“Sapienza”, University of Rome.

### 2.2. Instruments

To assess the development of the metalinguistic abilities of our participants, we used a range of tasks selected from an Italian metalinguistic ability test, called TAM-1 (*Test di abilità metalinguistiche n.1*) [40], validated on an Italian-speaking population. However, we did not use the original test but rather a selection of tasks with some adaptations of both the presentation modality and the target ages. While the TAM-1 is for children 4–6, our tasks target children from 5 to 7. This was due to our specific focus which addressed the transition from the last year of kindergarten to the first two years of primary school when all the studies reported in the classical literature on metalinguistic development highlight a significant transition (See Section 1).

An interesting point of the above tasks resides in the assessment criteria. Instead of a dichotomic modality, with only two possibilities (correct/incorrect), the coding system includes an intermediate level between an absent or incorrect response and a satisfactory one. This intermediate level suggests an incipient understanding of the metalinguistic principle relevant to the task, which, however, is not consistently applied. As a consequence, the coding of each item is based on a three-step scale: 0, 1, 2, where 0 matches no responses or incorrect responses; 1 matches partially correct responses, and 2 matches consistently correct responses. However, there is one task where this modality does not apply, namely Word Length, for reasons that will be explained further. In this study, we considered the following tasks:

**Word order** (from Ricciardelli, Rump, Proske [41], in turn, re-arranged from Pratt, Tunmer, Bowey, [42]) assesses children’s awareness of word order rules, in turn, based on the interconnection between word selection and combination rules. The task presents 9 sentences where canonical word order is upset due to increasingly complex transformations. The child is requested to overcome the conflicts generated by these transformations by re-establishing the correct syntactic order. Ex: *“Bananas are blue not*” (One transformation. Expected response: “Bananas are not blue”. “*The bird in the is tree*” (Two transformations. Expected response: “The bird is in the tree”). Scoring. 0: Responses coded as 0 are fundamentally elusive: the child does not respond or simply repeats the item or else reworks the global meaning of the item without operating on word order, as requested. (Ex: “Bananas are yellow”); 1: the child does operate on word order but either substitutes or adds some words, as he/she pleases (ex: “Bananas and apples are not blue”) or implements only one of the required changes when the item requires more than one (ex: “The bird in the tree is”); 2: the child can implement all the required transformations to re-establish the correct word order. **Max score: 18.**

**Word length** (from Ricciardelli, Rump, Proske [41], in turn, re-arranged from Papandropolou, Sinclair [43], and Berthoud-Papandropolou [18]) assesses children’s awareness of the distinction between form and referents in single words. The task presents 10 items where words of different lengths appear and the child is asked to say each time if the word is long or short. In some of these items, there is a conflict between the length of the word (alternatively long or short) and the dimensions of the corresponding referent (alternatively big or little). In others, the word (alternatively long or short) has no precise referent, and therefore there is no special conflict between form and content. Ex: “Train: is it a long or a short word?” (Expected response: “short”) “Strawberry: is it a long or a short word?” (Expected response: “long”) “Interesting: is it a long or a short word?” (Expected response: “long”). Scoring: for items with no precise referent, the scoring is 0/1, as there is no conflict between form and content. When this conflict exists, the two possibilities are 0 or 2, therefore with a higher score for the correct response, due to the difficulty to overcome the conflict. **Max score: 13**.

**Lexical segmentation** (from Pontecorvo, Tonucci, Zucchermaglio, [44]) assesses children’s awareness that sentences are built on discrete linguistic units that can be identified by clear-cut boundaries. The task presents 8 different sentences, increasingly longer, where the child has to identify and count the linguistic unities each sentence contains. The very detection of the boundaries between words might generate some conflict related to the exact point of the initial and final part of each word. Sentences are read aloud by the examiner but the child is allowed to look at their printed form on a sheet under his/her eyes. Ex: “The house is red”. (Expected word count: 4) “We drink coffee and milk for breakfast” (Expected word count: 7). Scoring. 0: the child unduly segments a single word into two parts (“break” and “fast” in breakfast”) or aggregates articles or prepositions with words (“wedrink” instead of “we” and “drink”); 1: the child segments nearly all the unities adequately but fails in some of them and therefore counts them incorrectly. Ex: “We drink coffee and milk for break/fast” (6 unities instead of 5). Alternatively, he/she may identify each of the linguistic unities correctly but recapitulates the total number of words incorrectly); 2. Responses are adequate on both grounds: correct identification and counting. **Max score: 16**.

**The rhyme task** (from Pontecorvo, Tonucci, Zucchermaglio [44]) assesses children’s awareness of the distinction between associations based on meanings (semantic) and associations based on sounds (phonetic). The task presents 8 items, and the child is requested to associate one word belonging to a given couple of words with a triad of other words on a purely phonetic basis. The items are organized in such a way that semantic associations enter into a conflict with phonetic associations, while the task requires keeping form and referent strictly separated. Ex: “If I say: pine, fine, line, what word fits better with these words: wine or tree? (expected response: “wine”). Scoring. 0: the child completely eludes the request and responds based on meaning instead of the form (Ex:, for the above item: “tree”, which matches the word “pine” based on meaning). 1: the child gives a correct response but the justification is based on a concrete instead than a phonetic criterion, as requested by the task. Ex: for the above item: “wine, because you can drink it”; 2: the child can associate words only based on the form, thus clearly separating form from meaning. **Max score: 16**.

**Symbol substitution** (from Ricciardelli, Rump, Proske [41], in turn, re-arranged form Ben Zeev [45]), assesses children’s awareness of the arbitrariness of word combination beyond conventional semantic and grammatical rules. The task presents 10 items where the child must simply substitute a given word with another in a regular sentence. The sentence that results from this substitution violates grammatical rules and produces both grammatical and semantic conflicts. Ex: “She swims well”. Let’s pretend that “she” is called “fishes” (expected response: “Fishes swims well”). Scoring. 0: Various forms of elusive responses. The child does not respond at all or repeats the item as is or declares the sentence is unacceptable. The child restructures the sentence with different words or adds other words in such a way as to give the sentence an acceptable overall meaning; 1: the child substitutes the proper word but in a different place of the sentence, again to make the sentence construction acceptable. Ex: for the item: “The cats are under the tree”, “under” must be replaced by “play” (expected response: “The cats are play the tree”). The child says: “The cats play under the tree”; 2: all the substitutions are implemented according to the requests. **Max score: 20.**

**Printed words, letters and number identification** (from Ricciardelli, Rump, Proske [41], in turn, re-arranged from Watson [46]) assesses children’s awareness of the identity and function of distinct types of signs. The task presents 9 stripes containing single letters, monosyllabic and multisyllabic words, in alternation with numbers, drawings, and a complete sentence. The child is asked to indicate linguistic units of different sizes following increasingly complex directions. Ex: stripe n.3 presents one capital letter (K), one lower-case letter (p), one two-digit number (37), one three digit-number (464), and one three-letter word (“dad”). The direction is: “Make a circle around the first letter of each word in this stripe”. (expected response: the child must circle the letter “d” of the only word present in this stripe, which is “dad”). Items are conceived in such a way that the child must overcome a double conflict: between heterogeneous categories of signs, and within the same category of the sign. Scoring. 0: the child circles several or all the units of each stripe, without distinction between letters, numbers, and words; 1: the child circles the entire word instead of the first letter; 2: The child applies the correct distinction between first letter and first word, as requested. **Max score: 18.**

**Maximum total ML score: 101.**

To test basic language abilities, we used a lexical comprehension test and a grammatical comprehension test drawn from the BVL 4–12 (*Batteria per la Valutazione del Linguaggio in bambini dai 4 ai 12 anni*) [47], an Italian battery for language assessment in children from 4 to 12, validated on an Italian-speaking population.

**Lexical comprehension**:

Preschool children. For every item (18 in all), the child must point to an image that matches the word uttered by the examiner (only substantive words) choosing between different images, one of which is a phonological distractor, another is a semantic distractor, and a third one is unrelated to the target. Scoring: 0 for correct and 1 for incorrect responses. **Max score: 18.**

Primary school children. The task follows the same modality as for preschoolers but presents a wider and more diversified range of words, for a total number of 42 items (31 substantive words, 10 verbs, and 1 adjective). The scoring follows the same criteria as in the task for preschoolers. **Max score: 42.**

**Grammatical comprehension**: The task is the same for preschool and primary school children. Children listen to a sentence uttered by the examiner (40 items) and must choose a picture that matches the meaning of the sentence between 4 alternatives, 3 of which are distractors, either on a morphological, syntactical or semantic basis. The scoring is also dichotomic. **Max score: 40.**

To measure nonverbal intelligence, we used the **Colored Raven’s Progressive matrices [48,49]**. The test measures general abstractive processes that are necessary to understand how semi-geometric patterns are constructed. The test presents 36 patterns of increasing complexity. The child must find a picture that matches the criterion underlying the construction of the vertical and the horizontal axis of the pattern, choosing between several alternatives. **Max score: 36**.

### 2.3. Procedures

The children were administered the tests by three psychologists who, at that moment, were participating in a seminar on developmental neuropsychology. They were intentionally selected for this study based on their training in the type of assessment used in our research. The administration took place in a quiet room of each school involved in the study and was subdivided into two sessions of 30 min each. In the first session, the Raven’s CPM and the two basic language ability tests were administered while the metalinguistic tasks were administered in the second session.

### 2.4. Data Analysis

To assess differences in metalinguistic abilities, we carried out an analysis of variance (ANOVA) on the metalinguistic scores by age group and gender with post hoc Gabriel tests (suitable for unequal group sizes). Familywise type I error rate was controlled using a Bonferroni adjusted *p*-level. As such, a real significance level (*p* < 0.05/48 = *p* < 0.001) was considered when interpreting the significance of pairwise comparisons. Pearson’s correlations were calculated to examine the bivariate relations between the 6 metalinguistic tasks, the CPM Raven’s test, and the two basic language ability tests. Lastly, an exploratory factor analysis examined the underlying structure of the metalinguistic abilities.

## 3. Results

### 3.1. Age-Group Differences in Metalinguistic Abilities

Table 2 reports the scores obtained by the three different age groups in all the meta-linguistic tasks (Mean, SD, SE).

**Figure 1:**
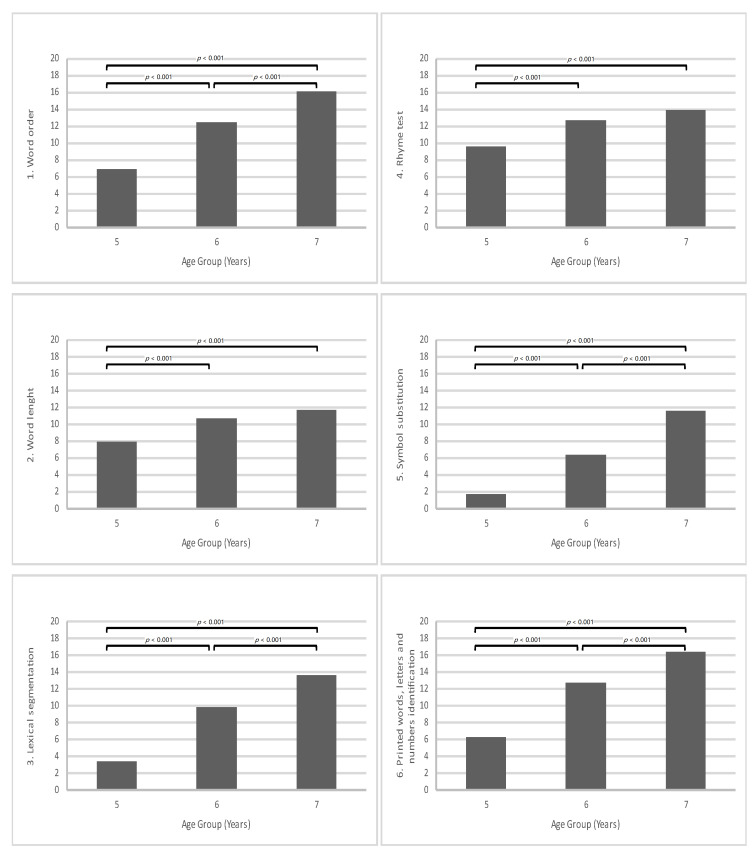
Age-group differences in metalinguistic abilities.

The ANOVA performed on these data showed statistically significant differences by age group in all tasks while differences related to gender were limited to only one case, and very marginally. As shown in Figure 1, the Word order score increased with age (F_2,154_ = 40.06; *p* < 0.001), with children in the 5–6-year-old group scoring lower than children in the 6–7-year-old and 7–8-year-old groups. The latter groups were also statistically different in Gabriel’s post hoc tests. There was a marginally significant effect of gender (F_1,157_ = 2.88; *p* = 0.092), with girls (M = 12.50) obtaining higher scores than boys (M = 12.50). The Word length score also increased with age (F_2,154_ = 26.54; *p* < 0.001). Post-hoc tests revealed that children in the 5–6-year-old group were significantly different from children in the 6–7-year-old and 7–8-year-old groups, while the latter groups did not differ statistically (Figure 1). The Lexical segmentation score increased with age (F_2,154_ = 59.75; *p* < 0.001), and all three age groups were significantly different from one another (Figure 1). The analysis of the Rhyme task revealed statistically significant group differences (F_2,154_ = 16.22; *p* < 0.001). As shown in Figure 1, children in the 5–6-year-old group scored lower than the two older groups, but these latter groups were not statistically different at post hoc tests. The analysis of Symbol substitution (F_2,154_ = 58.87; *p* < 0.001) and Printed words, letters and numbers identification (F_2,154_ = 38.06; *p* < 0.001) also revealed statistically significant differences by age. In the former case, all three age groups were statistically different from one another while, in the latter, children in the 6–7-year-old and 7–8-year-old groups did not differ significantly (Figure 1). In sum, for Word length, Rhyme task, and Printed words, letters and numbers identification there was a non-linear trend, while the scores of other subtests increased almost linearly with age (see Figure 1).

### 3.2. Associations between Metalinguistic Abilities, Language Abilities, and Nonverbal Intelligence

The 6 metalinguistic tasks were all positively interrelated (Table 3). The highest coefficient was observed between Lexical segmentation and Printed words, letters, and numbers identification. The lowest correlation was found between Word length and Rhyme, the two cases characterized by a non-linear trend by age in the previous analyses. Table 3 also reports the correlations between the metalinguistic scores with lexical and grammatical comprehension scores, and nonverbal intelligence scores, which were all positive and varying from moderate to large. Word order, Lexical segmentation, Symbol substitution, and Printed words, letters, and numbers identification presented a correlation with lexical comprehension greater than or equal to 0.65, which is a large magnitude effect. Word order, Lexical segmentation, and Symbol substitution were again the metalinguistic tasks that correlated the most with grammatical comprehension. In general, correlations between metalinguistic tasks and lexical comprehension showed larger coefficients than correlations between metalinguistic tasks and grammatical comprehension (Table 3)**.** Regarding the associations between metalinguistic tasks and the CPM Raven’s test, correlations were also found, except for the Rhyme task, although to a moderate degree.

### 3.3. The Structure of Metalinguistic Abilities

The 6 metalinguistic tasks were submitted to an exploratory factor analysis using a subsample of 123 children who had completed all the metalinguistic tasks. Despite the relatively small sample, the excellent result of the Kaiser-Meyer-Olkin test of sampling adequacy (KMO = 0.90) and the significance of Bartlett’s Test (Chi-square = 410.99 df =15 *p* < 0.001) demonstrated that the prerequisites for conducting the analysis were achieved. The Principal Axis Factoring method was applied as it is best suited for analyzing data from small samples.

The following eigenvalues were extracted: 3.98, 0.62, 0.49, 0.36, 0.32, 0.23. As only one eigenvalue was greater than 1.00 and the scree plot showed a flattening of the eigenvalue curve after the first eigenvalue, we decided to retain only the first principal axis, which explained 66% of the common variance in metalinguistic abilities. In decreasing factor loading order, we found Lexical segmentation (λ = 0.86), Printed words, letters and numbers identification (λ = 0.84), Word order (λ = 0.81), Symbol substitution (λ = 0.76), Word length (λ = 0.69), Rhyme task (λ = 0.62). These findings supported using a total battery score to have a global assessment of metalinguistic abilities. The estimated factor score representing this general factor was positively correlated with children’s age (r = 0.71), Lexical comprehension (r = 0.71), Grammatical comprehension (r = 0.54), and nonverbal intelligence (r = 0.55).

## 4. Discussion

In this study, we aimed to assess the gradual development of metalinguistic processes applied to constitutive aspects of language, in a range of tasks for children from 5 to 7 [1,4,10,16,25]. This age range was thought as particularly stimulating as it matches a significant turn in education, with the transition from the last year of kindergarten to the first year of primary school, and at the same time witnesses the growth of more general cognitive factors. To a sample of 160 native Italian-speaking and normally developing children from 5 to 7, we administered six metalinguistic tasks, a lexical comprehension test and a grammatical comprehension test, and a nonverbal intelligence test. These instruments were chosen to capture the relationships between metalinguistic abilities and more basic language abilities, as well as abstract cognitive and metacognitive capabilities [1].

It is to be noted that each metalinguistic task addresses some linguistic or semiotic principle of a very general and basic nature under the form of a given problem. For instance, sentences must follow a canonical word order so that words cannot be combined randomly (task n.1). The linguistic form is arbitrarily matched to referents in the physical world, which implies that form and referents must be treated on distinct planes (tasks n.2 and 4). Linguistic units are identifiable by clear-cut boundaries that must be respected to avoid semantic confusion (task n.3). Language is an arbitrary, combinatorial system that allows rearrangements of words if conventionally agreed (task n.5). Written words are distinct from other written signs, and also have a combinatorial nature, with single letters forming words and single numbers forming composite numbers (task n.6). The directions of our metalinguistic tasks trigger a series of grammatical, semantic, lexical, or semiotic conflicts, as it appears from the description of each task (See Section 2.2., above). Facing these conflicts requires detecting the components of the conflict itself, analyzing the differential weight of each component in relation to the specific direction of the task, and elaborating an explicit representation of the structural aspect under focus. All these processes are metalinguistic in nature, as they operate on language at a meta-level.

Back to the Word length task, our protocols provided suggestive examples of these different representations, some of which were under the requested meta-level while others were fully at that meta-level. For instance, a 5-year-old child, when requested to say if the Italian word ‘pasticcino’ (Eng: ‘cookie’) was long or short, declared it was a short one and spontaneously added: “It’s small, so sweet and it all stays in your mouth”. On the contrary, another child of the same age, after declaring ‘pasticcino’ was a long word, told the examiner: “Don’t you hear it? ‘Pas-tic-ci-no!’ It’s long! ” In a similar vein, a 6-year-old child said: “It’s a long word…, Can’t you hear it never ends, it’s not like ‘topo’ (Eng: ‘mouse’) because the *sound* is so long” (*Our italics*). While the first child remained at the concrete level of the referent (a cookie is a small object) the other children overcame the conflict between the representation of the referent and the representation of the form, and thus accessed a more abstract level, as manifested by their explicit justifications.

Using a two-factor ANOVA (age and gender) we showed that age was by far the main factor accounting for differences in metalinguistic performance. While there was a significant increase in all tasks with age, the pattern of growth across the three ages considered was partly different in some of the metalinguistic tasks. In Word length, Rhyme test, and Printed words, letters and numbers identification, there was a clear gap between the 5 year-olds, on the one hand, and the older children, on the other. We may interpret this marked transition as an indirect consequence of emergent literacy skills at the beginning of primary school, as expected. Indeed, the systematic practice of transferring sounds into written signs highly benefits the capability to treat form and content as separate entities (as required in Word length and Rhyme), and distinguish between different categories of written signs (as required in Printed words, letters, and numbers identification). Interestingly, after more than forty years since Berthoud-Papandropoulou’s emblematic study [18], our results in Word length showed some anticipation in the age of successful responses. Our 5 year-olds were more similar to Berthoud-Papandropoulou’s older children, and, in this respect, we might say that their ‘metalinguistic mind’ was more advanced.

In Word order, Lexical segmentation, and Symbol substitution, instead, the growth was quite linear, each age level yielding a significant increase. This more gradual growth does not contradict the relevance of the first transition between the 5 year-olds and the older children, which remains salient. Further factors, that seem more correlated to language acquisition processes than to specific literacy practices, are probably called into play [3,38,39]. Among these, we might consider an increased familiarity with canonical word order rules (as involved in Word order), with the use of longer utterances (as involved in Lexical segmentation), or with the flexible rearrangement of words in sentences (as involved in Symbol substitution) [21,22,23,24].

A second noteworthy result of our study was the high level of intercorrelations between metalinguistic tasks beyond their relative diversity. Thanks to these intercorrelations it has been possible to extract a single factor accounting for a large part of the common variance. We think this result may be attributed to the nature of the metalinguistic processes elicited by these tasks [1,10].

A third result was the association between metalinguistic abilities with basic lexical and grammatical competencies, and nonverbal intelligence processes, which proved positive in all cases, although to a different extent. Lexical comprehension was more strongly associated than grammatical comprehension with metalinguistic abilities, and the latter was only moderately correlated with nonverbal intelligence. This pattern of correlations suggests that metalinguistic abilities may be conceptualized as a construct midway between language and metacognitive abilities [1,10].

We think this study presents some elements of originality. The six metalinguistic tasks we used seem to capture core aspects of the capability to face the type of conflicts we outlined in children of the age range considered. These years can be seen as essential for the emergence of a ‘metalinguistic mind’. This result appeared partly from factor analysis, which showed the internal consistency of the tasks, and partly from the correlations with other abilities targeted for the same ages. Among the limitations, we will mention the reduced size of the sample and the fact that the tasks we used are not yet validated. Future research should take advantage of the promising results of the factor analysis to validate the tasks on a wider and fully representative sample to transform them into a test with precise age norms.

In addition, we believe our results can stimulate further studies on the developmental trajectories of atypical populations, such as those with neurodevelopmental disorders. Although these populations might present weaknesses at the basic language level, the range of linguistic profiles in neurodevelopmental disorders is so diversified that it is worth exploring the possibility that these children are also able to develop a ‘metalinguistic mind’ [37].

## Figures and Tables

**Table 1:** Distribution of age and gender groups in the sample.

	Girls		Boys		Total	
**Age Group**	***N***	**%**	***N***	**%**	***N***	**%**
5–6 years	25	15.8	27	17.1	52	32.9
6–7 years	32	20.3	31	19.6	63	39.9
7–8 years	16	10.1	27	17.1	43	27.2
**Total**	73	46.2	85	53.8	158	100.0

**Table 2:** Descriptive statistics for metalinguistic tasks by age group.

Tasks	Age Group	Mean	SD	SE
ML.1. Word order	5–6 years	6.91	5.80	0.79
	6–7 years	12.52	5.53	0.70
	7–8 years	16.12	3.19	0.49
ML.2. Word length	5–6 years	7.94	3.03	0.41
	6–7 years	10.68	2.68	0.34
	7–8 years	11.67	1.94	0.30
ML.3. Lexical segmentation	5–6 years	3.37	4.47	0.61
	6–7 years	9.79	5.87	0.74
	7–8 years	13.63	2.34	0.36
ML.4. Rhyme task	5–6 years	9.59	4.57	0.62
	6–7 years	12.67	3.97	0.50
	7–8 years	13.95	3.19	0.49
ML.5. Symbol substitution	5–6 years	1.72	2.78	0.38
	6–7 years	6.35	5.22	0.66
	7–8 years	11.58	4.91	0.75
ML.6. Printed words, letters and numbers	5–6 years	6.24	4.39	0.60
	6–7 years	12.68	4.50	0.57
	7–8 years	16.33	2.16	0.88

**Table 3:** Descriptive statistics and correlations.

Instrument	M	SD	1.	2.	3.	4.	5.	6.	7.	8.
ML.1. Word order	11.6	6.3								
ML.2 Word length	10.0	3.0	0.57 **							
ML.3. Lexical segmentation	8.7	6.2	0.71 **	0.59 **						
ML.4. Rhyme task	12.0	4.4	0.61 **	0.44 **	0.57 **					
ML.5. Symbol substitution	6.2	5.8	0.69 **	0.53 **	0.73 **	0.54 **				
ML.6. Printed words, letters and numbers	10.0	5.5	0.65 **	0.60 **	0.77 **	0.50 **	0.67 **			
BVL. Lexical comprehension	22.8	9.1	0.65 **	0.56 **	0.65 **	0.46 **	0.70 **	0.68 **		
BVL. Grammatical comprehension	28.5	8.9	0.52 **	0.38 **	0.53 **	0.44 **	0.54 **	0.46 **	0.65 **	
Raven’s CPM	20.4	5.8	0.42 **	0.42 **	0.46 **	0.26 **	0.49 **	0.36 **	0.54 **	0.59 **

Total *N* = 160; ** *p* < 0.001. Legend: ML: Metalinguistic; BVL: Battery for language assessment; Raven’s CPM: Raven’s Colored Progressive Matrices.

## Data Availability

Not applicable.
